# Online Compensation of Systematic Effects in Stimuli Generation for XR-Based SSVEP BCIs

**DOI:** 10.3390/s26030766

**Published:** 2026-01-23

**Authors:** Leopoldo Angrisani, Egidio De Benedetto, Matteo D’Iorio, Luigi Duraccio, Fabrizio Lo Regio, Annarita Tedesco

**Affiliations:** 1 Department of Electrical Engineering and Information Technology (DIETI), University of Naples Federico II, Via Claudio n.21, 80125 Naples, Italy; angrisan@unina.it (L.A.); matteo.diorio@unina.it (M.D.); luigi.duraccio@unina.it (L.D.); fabrizio.loregio@unina.it (F.L.R.); 2 Department of Public Health, University of Naples Federico II, Via Pansini, 80131 Naples, Italy; annarita.tedesco@unina.it

**Keywords:** Brain–Computer Interface (BCI), Extended Reality (XR), Filter Bank Canonical Correlation Analysis (FBCCA), measurement, online compensation, Steady-State Visually Evoked Potential (SSVEP), systematic effects, wearable systems

## Abstract

**Background**: Brain–Computer Interfaces (BCIs) based on Steady-State Visually Evoked Potentials (SSVEPs) and Extended Reality (XR) offer promising solutions for highly wearable applications, but their classification performance can be affected by systematic effects in stimulus presentation. **Novelty**: This study introduces a novel online compensation method to compensate for systematic effects in the Refresh Rate (RR) of XR displays, enhancing SSVEP classification without requiring additional training or invasive measurements. **Methods**: A non-invasive monitoring module was incorporated into the developed BCI pipeline to measure frame rate variations in the XR display, allowing deviations between nominal RR and measured values to be automatically detected and compensated for. Classification performance was evaluated using Filter Bank Canonical Correlation Analysis (FBCCA). Statistical significance was assessed using Student’s *t*-test. **Materials**: Two datasets were used: a dataset based on Moverio BT-350, including 9 subjects, and a dataset based on HoloLens 2, including 30 subjects, all collected by the authors. **Results**: The proposed compensation method led to significant improvements in SSVEP classification accuracy, proportional to the magnitude of fps deviations. In some cases, classification accuracy increased by up to 300% relative to its original value. Statistical analyses confirmed the reliability of the results across subjects and datasets. **Conclusions**: These findings show that the proposed method effectively enhances SSVEP-based BCIs in XR environments and provides a robust foundation for practical applications requiring high reliability.

## 1. Introduction

Brain–Computer Interfaces (BCIs) have increasingly emerged as innovative technologies enabling intuitive and direct communication between the human brain and external systems [1,2,3,4]. By measuring brain activity, BCIs translate voluntary and involuntary neural modulations into actionable commands, thus allowing users to interact seamlessly with digital or physical environments [5]. BCIs can be primarily categorized according to the technique used to measure brain signals, such as functional magnetic resonance imaging (fMRI), magnetoencephalography, near-infrared spectroscopy, or electroencephalography (EEG) [6]. According to the state of the art, EEG is considered the most effective technique for daily-use wearable BCI applications, due to its non-invasiveness, low cost, and ease of implementation [7].

Among the different BCI paradigms, Steady-State Visually Evoked Potential (SSVEP) is one of the most widely explored [8,9,10]. SSVEPs are brain oscillatory responses generated in the primary visual cortex when a subject is exposed to flickering visual stimuli, typically within the frequency range of 1 to 100 Hz [11]. These responses exhibit a strong correlation with the stimulation frequency and its harmonics [12], enabling robust frequency tagging for decoding user intent. SSVEP-based BCIs have become a reference paradigm for reliable and efficient brain signal classification [13] due to their high signal-to-noise ratio (SNR), particularly within the frequency range of 8 to 20 Hz [14].

Traditionally, SSVEP stimuli are presented on a computer screen (CS), most commonly an LCD monitor positioned in front of the user [15]. This configuration, which can display up to 200 different stimuli simultaneously [16], has proven highly effective for applications such as the *BCI Speller*, a system that enables individuals with severe motor impairments to communicate through brain activity alone, without requiring any muscular control [17,18]. Despite its effectiveness and widespread adoption as a benchmark setup, this configuration is inherently bulky and therefore restricts the portability of BCIs. Consequently, SSVEP-based BCIs have remained largely confined to controlled or laboratory environments [19].

To overcome these limitations, Extended Reality (XR) Head-Mounted Displays (HMDs) have recently emerged as wearable and ergonomic devices capable of delivering visual stimulation in highly interactive and immersive environments [20]. By eliminating the need for bulky and fixed workstations, XR HMDs offer greater portability and user comfort [21]. Consequently, these devices are progressively replacing traditional CS setups [22], thus expanding the applicability of SSVEP-based BCIs to everyday and mobile contexts.

However, despite these advantages, XR HMDs face a significant limitation in stimulus generation, primarily due to hardware constraints—their Refresh Rate (RR), defined as the number of times per second the display updates the image on the screen, may deviate from the nominal value specified by the manufacturer and, more importantly, it may not be constant over time [23]. If the actual RR deviates from the assumed value, the generated stimuli will shift proportionally from their intended frequencies [24]. This leads to an incorrect classification of the visual stimuli actually perceived by users, thereby affecting the overall performance of SSVEP-based BCIs [25].

From a metrological perspective, RR issues arise from a combination of systematic and random effects. Systematic effects correspond to persistent deviations between the actual RR of the XR HMD and its nominal value, whereas random effects originate from fluctuations in computational load during the application runtime and are responsible for short-term variations in the RR (such random effects are known as jitter [26]). As stated by the *Guide to the Expression of Uncertainty in Measurements* (GUM) [27], random effects cannot be compensated for by design, as they are inherently unpredictable, while systematic effects can be reliably identified and compensated for [28]. Nevertheless, despite various methods having been proposed, there is currently a lack of strategies that enable online compensation. Offline calibration approaches, which estimate the device’s average RR and compare it to the nominal value, e.g., using photodiodes [29], are insufficient, as the average RR can deviate significantly during each session, invalidating any static compensation determined at the beginning of the experiments. As a result, current research generally assumes a fixed RR equal to the nominal value specified by the XR HMD manufacturer, leaving systematic RR deviations uncorrected and thus increasing the risk of misclassifications.

Starting from these considerations, this paper introduces a novel measurement method for the online compensation of systematic effects in stimulus generation for XR-based SSVEP BCIs. The proposed approach relies on the monitoring of a quantity closely related to, but distinct from, the RR, namely the frame rate (frames per second, fps), defined as the number of frames generated per second by the system [30]. Although the fps rate is primarily determined by the software application, monitoring its variations provides indirect and online information about the hardware behavior of the XR device and, consequently, about the RR, which is responsible for deviations in the generated flickering stimulation. To evaluate the robustness of the proposed method across different hardware configurations, this study analyzes two XR-based SSVEP BCI setups [19,31], based on XR devices with distinct technical specifications.

The paper is organized as follows. In Section 2, a background of XR-based SSVEP-based BCIs is provided. Section 3 describes the proposed method. In Section 4, the case studies and the results are described in detail. Finally, conclusions are drawn, and future work is outlined.

## 2. Background

**Figure 1:**
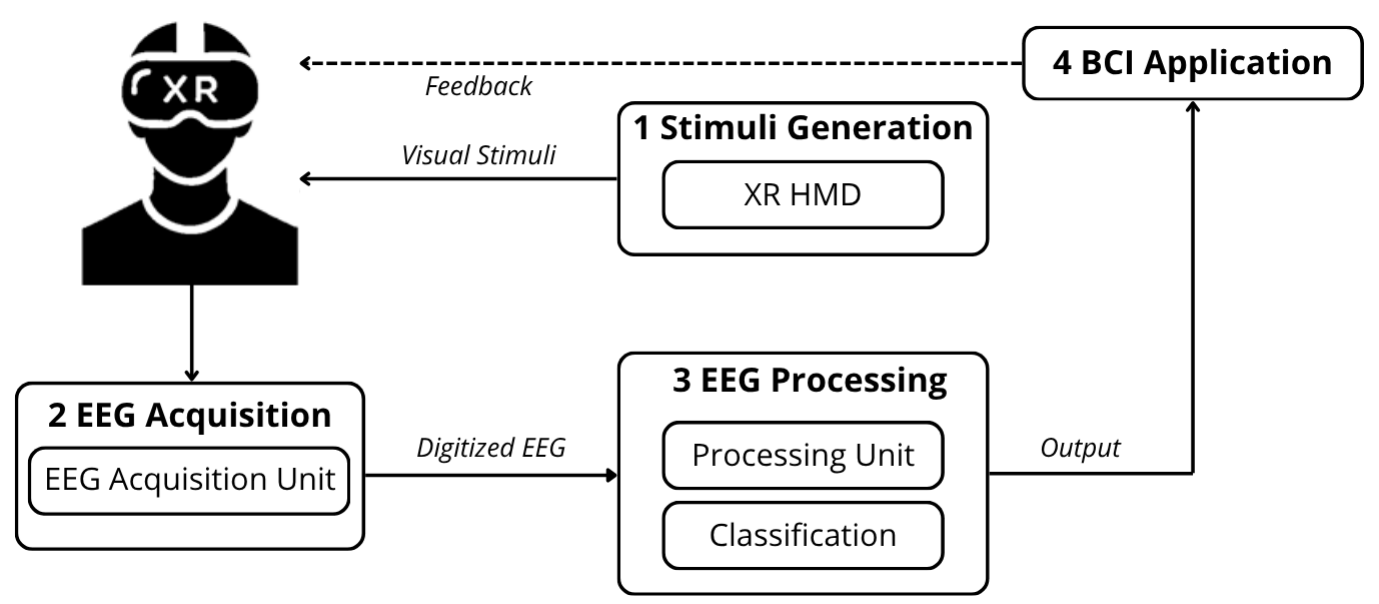
General architecture of XR-based SSVEP BCIs.

The typical architecture of an XR-based SSVEP BCI is illustrated in Figure 1. This system is generally composed of four main functional blocks.

The first block, *Stimuli Generation*, involves the use of an XR HMD to present *N* concurrent visual stimuli within the user’s field of view (FoV). Each stimulus flickers at a distinct frequency, allowing the system to associate each stimulus with a specific command. The XR HMD offers an immersive and highly customizable visual experience, directly integrating with the second block, i.e., the *EEG Acquisition* unit. This consists of a portable EEG acquisition system that measures the user’s brain activity in response to the presented stimuli. The number and typology of EEG channels can vary according to the specific requirements of wearability and signal quality, balancing usability with performance.

Once acquired, the digitized EEG signals are processed by the *EEG Processing* unit (Block #3). This unit may either be within the *EEG Acquisition* unit, or be external, i.e., connected to a laptop or the XR HMD itself through wireless communication. It performs operations on the EEG signals, such as filtering and feature extraction, and runs the dedicated classification algorithm to recognize which stimulus has been observed by the user. In fact, given *N* flickering stimuli, the recognition of the stimulus the user focused on is viewed as a N-class classification problem [32]. Classification is successful when the output of the classifier corresponds to the actual visual stimulus observed by the user.

Finally, the fourth block, *BCI Application*, receives the classified output and executes the corresponding action. Typically, a feedback mechanism is implemented, often integrated into the XR application, to confirm the user’s selection [33].

### 2.1. Digital Rendering of the Flickering Stimuli

Flickering stimuli are rendered by alternating frames with different light intensities, which are displayed sequentially on the XR HMD. A general approach to encode a frequency *f* into this frames alternance is through a modulation function, designed to vary the pixel intensity *p* within a normalized range [0,1]. The pixel intensity *p* is given by

(1) $$p=\frac{1}{2}\left\{1+wave\left(f,i,RR\right)\right\},$$

where wave(·) represents a general periodic wave function, RR is the intended Refresh Rate of the device, and *i* is the frame index. The normalized levels of pixel intensity [0,1] are mapped onto the display’s output such that 0 corresponds to the device’s minimum luminance and 1 to its maximum. In grayscale representation, the intensity varies continuously from black (p=0) to white (p=1), with intermediate values producing proportionally scaled gray levels. For color stimuli, the same modulation is applied independently to each of the red, green, and blue (RGB) channels.

The function wave(·) can assume different forms depending on the desired flickering pattern. A first approach employs a square-wave function square[2πf(i/RR)], which alternates between two discrete intensity levels, i.e., p=0 and p=1, at a fixed frequency [34]. An alternative approach for generating the flickering stimulus is the sinusoidal method [18], which provides a smooth and continuous transition within the normalized range [0,1]. In this case, the corresponding wave(·) function is a sinusoidal waveform with phase ϕ, expressed as sin[2πf(i/RR)+ϕ]. To the best of the authors’ knowledge, there is no experimental evidence in the literature indicating that the waveform type affects the susceptibility of RR fluctuations. Therefore, from this perspective, both stimulus generation methods can be used.

**Figure 2:**
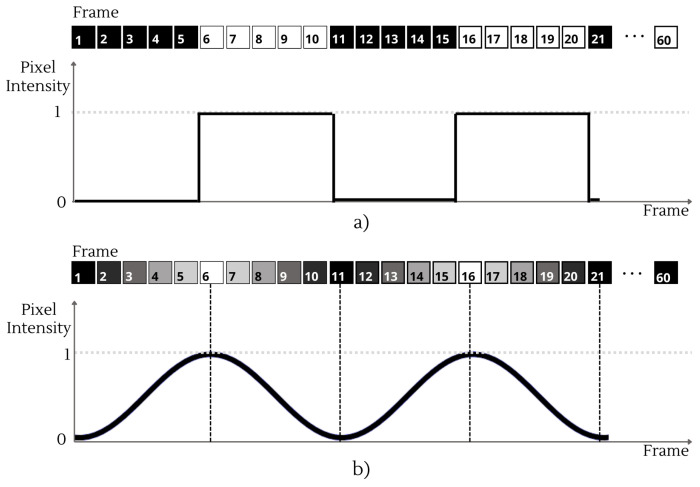
Representation of a 6 Hz flickering stimulus in grayscale on a display with a Refresh Rate of RR=60 Hz over a 60-frame window: (**a**) square-wave modulation, where each cycle alternates between black and white frames; (**b**) sinusoidal-wave modulation, where each cycle includes black, white, and intermediate gray frames.

Figure 2 illustrates a comparison between the square-wave and sinusoidal modulation methods for a stimulation frequency of 6 Hz and an intended Refresh Rate of RR=60 Hz.

### 2.2. Refresh Rate Instability

The effectiveness of rendering the flickering stimuli is constrained by the architecture of the XR HMD.

From (1), a flickering stimulus at frequency *f* is represented as a pattern of frames, indexed by i=1,…,k, that repeats periodically on a display with a RR typically specified by the manufacturer, here denoted as the nominal value $RR_{n}$. However, as previously noted, XR HMDs generally exhibit both systematic and random effects that result in an instability of the actual RR, here denoted as $RR_{a}$, leading to deviations in the delivered flickering stimulus [23,35].

Under the nominal specification $RR_{n}$, the stimulus flicker frequency *f* is realized by selecting an integer cycle length $k=RR_{n}/f$. However, at runtime, the stimulus actually flickers at $f_{a}=RR_{a}/k$, so that any deviation of the actual Refresh Rate $RR_{a}$ from its nominal value $RR_{n}$ results in $f_{a}\neq f$. Consequently, variability in the Refresh Rate produces a frequency mismatch in the delivered stimulus [24], with the actual flickering frequency $f_{a}$ expressed as

(2) $$f_{a}=f\frac{RR_{a}}{RR_{n}}.$$

**Figure 3:**
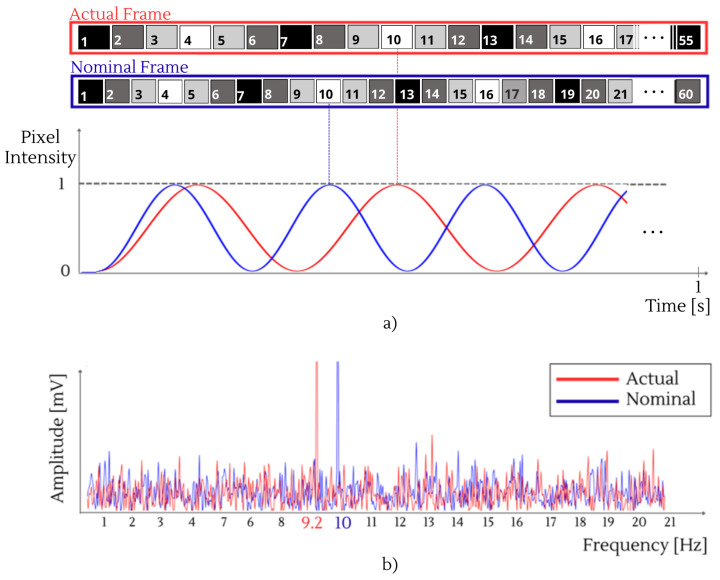
Comparison between the nominal and actual rendering of a 10-Hz visual stimulus when the display RR decreases from $RR_{n}=60$ Hz to $RR_{a}=55$ Hz: (**a**) Temporal evolution of the pixel intensity (sine wave modulation) in relation to the frame updates. (**b**) Frequency spectrum showing the shift of the EEG peak in frequency from the nominal 10.0 Hz (blue) to the actual 9.2 Hz (red).

The deviation of flickering frequency from *f* to $f_{a}$ can have significant consequences for the classification of SSVEPs. As an example, consider an XR device with $RR_{n}$ of 60 Hz, intended to render flickering stimuli at 9.0 Hz and 10.0 Hz, corresponding to commands A and B, respectively. If the RR decreases to $RR_{a}$ = 55 Hz, these stimulus frequencies would be shifted to 8.3 Hz and 9.2 Hz. Consequently, if the user’s EEG exhibits a spectral peak around 9 Hz, it becomes ambiguous whether the user was attending to the stimulus originally designed to flicker at 9.0 Hz (command A) or to the one initially set at 10.0 Hz (command B) that was downshifted to 9.2 Hz due to the RR reduction. This discrepancy introduces a classification error, leading the system to associate an incorrect command instead of the one the user originally intended. A similar issue occurs when $RR_{a}>RR_{n}$, which causes an upward shift in the frequencies. For completeness, Figure 3 provides an illustrative example of the impact of RR deviations for a 10-Hz visual stimulus. As illustrated in Figure 3a, a lower refresh rate results in longer frame durations, leading to a slower temporal evolution of the intensity of the rendered visual stimulus. This deviation effectively downshifts the flickering frequency and generates a cumulative phase delay, which is clearly visible in the misalignment of the sine waves over time. The resulting spectral shift is further highlighted in the frequency domain in Figure 3b.

As mentioned in Section 1, RR variability in XR HMDs arises from a combination of systematic and random effects. Systematic effects produce consistent deviations of the actual RR from its nominal value, often occurring when the computational demand of the XR application approaches or exceeds the available processing capacity. Random effects, in contrast, originate from unpredictable influences, such as transient fluctuations in computational load or environmental conditions (e.g., user motion or ambient lighting), which cause short-term variations in RR over time. While random effects are inherently unpredictable and cannot be addressed by design, systematic effects can be reliably identified and comprensated for [28]. Nevertheless, at the state of the art, strategies for online compensation of these systematic effects are still lacking.

## 3. Method

**Figure 4:**
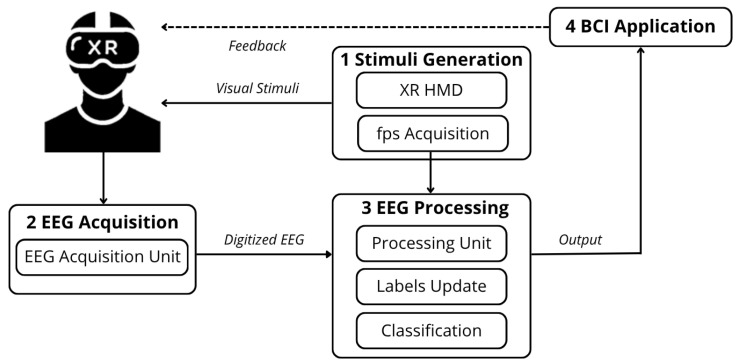
Proposed architecture of XR-based SSVEP BCI with online compensation of Refresh Rate fluctuations.

The proposed method introduces a novel enhancement to the architecture of an XR-based SSVEP BCI, previously illustrated in Figure 1. The traditional architecture is enhanced by the addition of two functional units: one responsible for monitoring fps variability (*fps acquisition*) and another for updating the labels of the rendered frequencies (*label update*). This results in the new architecture, depicted in Figure 4. As aforementioned, monitoring the fps enables software-based, online assessment of RR variability, as these two quantities are directly correlated [36]. In fact, while the RR indicates the rate at which the display presents frames, the fps corresponds to the rate at which the Graphics Processing Unit (GPU) generates and delivers them.

The novel pipeline for XR-based SSVEP BCIs is described as follows:

1. The XR HMD renders *N* flickering stimuli at nominal frequencies $f_{1},f_{2},\ldots ,f_{N}$ under the nominal Refresh Rate $RR_{n}$; the nominal label set $L_{n}={\left\{f_{n}\right\}}_{n=1}^{N}$ is defined for the flickering stimuli.
2. Within each stimulation window, EEG signals are acquired, along with the timestamp of each rendered frame [$t_{1},t_{2},\ldots ,t_{M}$], and the corresponding Refresh Rate $RR_{a}$ for that window is subsequently computed according to (3):
  (3) $$RR_{a}=\frac{1}{M}\sum_{m=2}^{M}\frac{1}{t_{m}-t_{m-1}}$$
  where the term *M* represents the number of frames generated by the XR HMD during the stimulation window (Accordingly, the actual refresh rate is estimated starting from computing the mean of the XR HMD frame timestamps. While higher-order statistics, such as variance, could be included to explicitly account for jitter effects, this aspect is beyond the scope of the present study and is left for future investigations).
3. The actual Refresh Rate $RR_{a}$ and the nominal frequency labels ${\left\{f_{n}\right\}}_{n=1}^{N}$ are used to compute the actual flickering frequencies through (2), yielding the actual label set $L_{a}={\left\{f_{a_{n}}\right\}}_{n=1}^{N}$.
4. The EEG signals for each stimulation window, along with the actual label set $L_{t}$, are forwarded to *EEG Processing*. Hence, the classification unit produces a decision. A correct classification occurs when the frequency identified by the classification unit corresponds to the stimulus frequency observed by the user.

The proposed method does not rely on a specific XR HMD or display technology. As long as the application can access frame-timing information (a capability standard in modern XR HMDs), the method can be applied without modification. Within the engine used for XR application development, such as *Unity* [37] or *Unreal* [38], platform function packs can be employed to collect the frame timestamps. Importantly, the method does not require integration of any external sensors, simplifying the setup while preserving the ergonomics and wearability of the XR device, which are essential for the implementation of daily-use, online XR-based SSVEP BCIs.

## 4. Experimental Validation

To evaluate the effectiveness of the proposed method relative to traditional approaches, the pipeline described in Section 3 was implemented in two distinct experimental setups. Specifically, two SSVEP datasets, each acquired using a different XR HMD, were considered for comparison.

### 4.1. Description of the Datasets

With regards to the stimuli generation, the two considered datasets employed the Epson Moverio BT-350 [19] and the Microsoft HoloLens 2 [31], two off-the-shelf optical see-through XR HMDs. Although the two XR HMDs are no longer state-of-the-art, their behavior remains of considerable interest for identifying possible deviations in the RR, an issue that may also occur in more recent XR HMDs, and for enabling the implementation of corrective actions.

These datasets provided EEG recordings along with time stamps, enabling the estimation of the effective display Refresh Rate. Both SSVEP BCI systems are ideal candidates for daily-use BCI applications relying on highly integrated and wearable hardware configurations.

- The Epson Moverio BT-350 features a diagonal FoV of 23° and a nominal RR of 30 Hz. The XR application was developed via *Android Studio* and configured to display a 2 × 2 matrix consisting of *N* = 4 visual stimuli. These stimuli were flickered at fixed frequencies of 8 Hz, 10 Hz, 12 Hz, and 15 Hz, implemented through a square wave modulation between black and white. EEG signals were acquired using a single-channel, differential setup using the [39] device, a 10-bit Analog-to-Digital Converter (ADC) operating at a sampling rate of 256 samples per second. Two active, dry electrodes were placed on the subjects’ scalp in *Oz* and *Fz*, while a passive reference electrode was placed in *A2*, according to the 10–20 International System [40]. Experiments involved 9 healthy adult volunteers with normal or corrected-to-normal vision. Each subject was instructed to focus on one of the four visual stimuli for a duration of 10 s. The procedure was repeated across five trials for each stimulus, resulting in a total of 20 signals per subject. Although the number of subjects may appear limited, it is consistent with the sample sizes commonly adopted in many experimental setups in XR-based SSVEP research [41,42,43,44], and it does not compromise the reliability or validity of the results presented.
- Microsoft HoloLens 2 is characterized by a diagonal FoV of 52° and a nominal RR of 60 Hz. The stimulation was developed using *Unity* [37]. A total of N=8 stimuli, placed within a 2 × 4 matrix, flickered at frequencies ranging from 8 Hz to 15 Hz in 1 Hz steps according to the sinusoidal waveform method, in grayscale. EEG signals were recorded at a sampling rate of 250 Hz using the g.tec *Unicorn Hybrid Black* device [45]. Wet electrodes were placed on the subject’s scalp over the occipital region, specifically at sites *O7*, *PO3*, *POz*, *PO4*, *PO8*, *O1*, *Oz*, and *O2*, in accordance with the international 10–20 system [40]. Additionally, two wet electrodes were positioned on the subject’s mastoids, serving as the reference and ground electrodes. EEG signals were recorded from 30 healthy adult volunteers, each performing 5 acquisition cycles consisting of the 8 visual stimuli, resulting in a total of 40 EEG recordings per subject.

EEG signals from both datasets were windowed over the first 1.25 s of each trial, which represents the typical time interval adopted in online SSVEP-based BCIs and carrying the maximum informative contribution of the response [46]. In Table 1, the features of two datasets are shown for comparison.

### 4.2. Classification Algorithm

The EEG classification was performed using the Filter Bank Canonical Correlation Analysis (FBCCA) [22], an enhanced version of the traditional Canonical Correlation Analysis (CCA) [47], and consists of three main steps:

1. First, the original EEG signal *X* is decomposed into a set of *S* sub-band components $X_{s}$ (with s=1,…,S) by means of a bank of band-pass filters with distinct passbands. This step aims to extract independent information embedded in both the fundamental and harmonic components of the SSVEPs.
2. Then, for each stimulation frequency $f_{n}$, a set of sinusoidal reference signals $Y_{fn}$ is constructed by including $N_{h}$ harmonics, as follows:
  (4) $$Y_{fn}=\left[\begin{array}{c} \sin(2\pi \cdot 1\cdot f_{n}\;t)\\ \cos(2\pi \cdot 1\cdot f_{n}\;t)\\ \vdots \\ \sin(2\pi \cdot N_{h}\cdot f_{n}\;t)\\ \cos(2\pi \cdot N_{h}\cdot f_{n}\;t) \end{array}\right]$$
  CCA is then applied to each pair $\left(X_{s},Y_{fn}\right)$, yielding correlation coefficients $\rho_{n}^{s}\in \left[0,1\right]$ for each *n*-th frequency and each *s*-th sub-band.
3. Finally, a weighted sum of the squared correlation coefficients $\rho_{n}^{s}$ across all sub-bands (i.e., $\rho_{n}^{1},\ldots ,\rho_{n}^{S}$) is computed for stimulus identification:
  (5) $${\tilde{\rho }}_{n}=\sum_{s=1}^{S}w\left(s\right)\cdot {\left(\rho_{n}^{s}\right)}^{2}$$
  where the weights are defined as $w\left(s\right)=s^{-a}+b$, with *a* and *b* being constant parameters. The frequency corresponding to the maximal ${\tilde{\rho }}_{n}$ is identified as the frequency of the flickering stimulus perceived by the user.

Before comparing the performance of the traditional classification method with that of the proposal, a Leave-One-Subject-Out (LOSO) validation procedure was employed to determine the optimal configuration of the parameters {*S*, $N_{h}$, *a*, *b*}, thereby simulating an online scenario. Specifically, in each fold, one subject was held out for validation, while the remaining N−1 subjects were used as the training set. For the current fold, a grid search was conducted on the training data to identify the parameter combination yielding the highest mean classification accuracy across the N−1 subjects. The grid search explored the following ranges: *S* and $N_{h}$ from 1 to 4 with a step size of 1; *a* in the range [0,2] with a step size of 0.25; and *b* in the range [0,1] with a step size of 0.25. The optimal parameters obtained from the training phase were then applied to the validation subject, thus emulating an online application scenario.

For each dataset, the procedure was performed twice: first using the static frequency labels $f_{n}$ (traditional method), and then using the dynamically updated labels $f_{a}$ (proposed method). The performance evaluation was expressed in terms of classification accuracy *A*, defined as the ratio between the number of correctly classified stimuli $N_{C}$ and the total number of stimuli *N*, as given by the following equation:

(6) $$A=\frac{N_{C}}{N}\cdot 100\;\;\left[\%\right].$$

Overall, the main advantage of using FBCCA over more complex machine learning models is that FBCCA is fundamentally a signal processing method. When combined with the selected LOSO CV strategy, it minimizes the risk of underfitting and enables reliable performance without the need for large datasets.

### 4.3. Experimental Results

**Figure 5:**
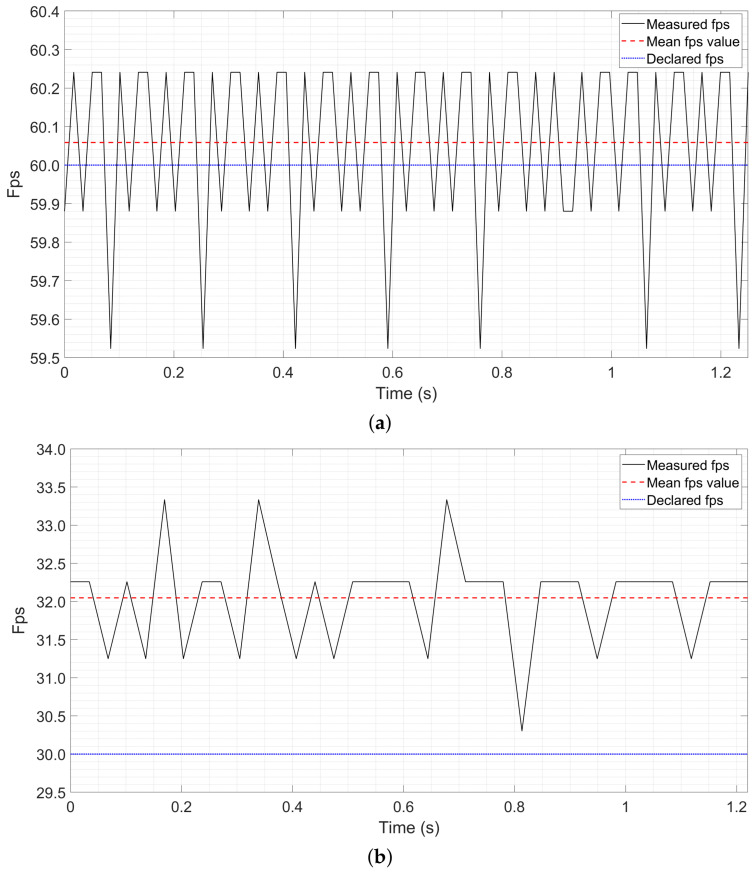
Measured frame rate (fps) during a stimulation phase along with its mean value and the declared value as provided by the manufacturer, for HoloLens 2 (**a**) and for Moverio BT-350 (**b**).

In Figure 5a,b, examples of the acquired fps during a stimulation phase are shown for the Microsoft HoloLens 2 and the Epson Moverio BT-350, respectively.

As can be observed, temporal fluctuations in the frame rate arise from random (i.e., jitter) effects, whereas the mismatch between the mean frame rate and the manufacturer-specified nominal Refresh Rate ($RR_{n}$) reveals a systematic deviation. Specifically, for the HoloLens 2, the measured Refresh Rate $RR_{a}$ (i.e., the mean value of the acquired fps) deviates slightly from the nominal $RR_{n}$ of 60 Hz, reaching 60.06 Hz (Notably, this deviation falls within the uncertainty range associated with the resolution at which $RR_{n}$ is provided by the manufacturer). In contrast, for the Moverio BT-350, the deviation is more pronounced, with the mean Refresh Rate increasing from 30 Hz to 32 Hz. Another noteworthy aspect is the presence of jitter (with frame rate fluctuations ranging from 30.5 to 33.5 fps), which clearly indicates the influence of random effect. In relative terms, the percentage deviation is approximately 0.1% for the HoloLens 2 and about 6.7% for the Epson Moverio BT-350.

Classification accuracy was evaluated by performing the traditional method, i.e., using the static frequency labels, and the proposed method, i.e., updating the labels according to the acquired fps. In Table 2 and Table 3, results in terms of mean classification accuracy are shown for each subject for HoloLens 2 and Moverio dataset, respectively. The average performance for both methods was assessed by evaluating the mean selection accuracy across all the subjects along with the corresponding standard uncertainty, according to a type-A evaluation [27].

With regards to Microsoft HoloLens 2, as shown in Table 2, both methods yield comparable performance, with an average value of (71.2±4.0)% for the traditional method and (71.1 ± 3.8)% for the proposed method. This outcome is attributable to low deviation between the actual RR and the nominal value provided by the manufacturer. Conversely, the Moverio dataset (Table 3) reveals a substantial difference between the two approaches. As a matter of fact, the proposed adaptive method significantly outperforms the traditional one, increasing the classification accuracy from (23.3 ± 2.6)% to (45.0 ± 7.5)%. The systematic shift shown in Figure 5b strongly affected the elicited stimulation frequencies, causing the traditional approach to degrade toward near-random classification performance. By contrast, the proposed method effectively compensates for this shift, leading on average to a doubling of classification accuracy. Although the achieved performance is not yet sufficient to ensure fully reliable operation for this specific system configuration, the results clearly demonstrate that the proposed compensation strategy represents a substantial and meaningful improvement in system performance.

This significant improvement highlights the robustness of the proposed method when dealing with devices characterized by unstable RR, as observed with the Moverio. In such a scenario, the static frequency labels become unreliable, whereas the adaptive method effectively compesates for these variations by updating the labels according to the real-time fps. To rigorously validate the discrepancies between the traditional and the proposed method, further analysis was performed using the Student’s *t*-test [48]. In particular, for both the case studies, the presence of a significant difference was assessed by comparing the two accuracy vectors. The significance level α was set equal to 0.05. While for the HoloLens 2 dataset, the test does not lead to the rejection of the null hypothesis (p>0.99), which considers the data originated from the same probability distribution, the results for the Moverio dataset (p<0.01) indicated that the performance obtained by the proposed method was significantly better than that of the traditional method. It follows that, while the difference for HoloLens 2 is not statistically significant, the improvement achieved by the adaptive method on the Moverio dataset is statistically significant. These results confirm that the proposed method’s effectiveness is highly correlated with the extent of the RR deviation from its nominal value.

With the aim of further enhancing the reliability of SSVEP classification, future research will also seek to mitigate the impact of random effects (i.e., jitter effects). These aspects were not considered in the present study, but their inclusion is expected to further strengthen the robustness of XR-based SSVEP BCI systems.

## 5. Conclusions

This study focused on improving the classification accuracy of SSVEPs in highly wearable BCIs operating within XR environments. The enhancement was obtained by introducing an adaptive procedure capable of compensating for systematic deviations that occur during the generation of visual stimuli used for SSVEP elicitation. Specifically, a dedicated monitoring module was incorporated into the XR-BCI architecture to continuously measure the actual frame rate of the stimulus rendering system. By comparing the measured values with the nominal RR of the XR display, the module enables the identification and correction of frequency deviations that could otherwise impair the reliability of the elicited SSVEP responses. Two experimental case studies were considered to evaluate the effectiveness of the proposed solution. The findings show that the classification performance increases proportionally to the extent of the detected frame rate variation. In some cases, classification accuracy increased by up to 300% relative to its original value. Statistical evaluation further supports the role of the proposed compensation strategy as a key enabler for achieving consistent operation of XR-based SSVEP BCIs, even under non-ideal display conditions.

## Figures and Tables

**Table 1:** Features of the datasets used for experimental validation of the proposed method.

Features	Dataset	
Literature Source	Arpaia et al. [19]	Angrisani et al. [31]
XR HMD used	*Epson Moverio BT-350*	*Microsoft HoloLens 2*
Nominal RR [Hz]	30	60
Diagonal FoV [°]	23	52
EEG device	Olimex EEG-SMT	Unicorn Hybrid black
EEG channels	Oz	O7, PO3, POz, PO4, PO8, O1, Oz, O2
EEG sampling rate [sps]	256	250
Stimulation frequencies [Hz]	{8,10,12,15}	{8,9,10,11,12,13,14,15}
Stimulation waveform	Square	Sinusoidal
No. Subjects	9	30
Acquisition Cycles	5	5
Signals per subject	20	40

**Table 2:** Classification Accuracy for HoloLens 2 Dataset in terms of mean and standard uncertainty across all the subjects.

Method/Subject	Traditional Method	Proposed Method
1	12.5%	17.5%
2	75.0%	72.5%
3	92.5%	90.0%
4	87.5%	87.5%
5	45.0%	45.0%
6	80.0%	82.5%
7	77.5%	75.0%
8	67.5%	65.0%
9	77.5%	75.0%
10	67.5%	65.0%
11	92.5%	95.0%
12	80.0%	80.0%
13	87.5%	90.0%
14	77.5%	82.5%
15	67.5%	70.0%
16	100.0%	97.5%
17	80.0%	77.5%
18	95.0%	95.0%
19	100.0%	100.0%
20	95.0%	92.5%
21	65.0%	65.0%
22	70.0%	65.0%
23	30.0%	35.0%
24	32.5%	37.5%
25	52.5%	52.5%
26	77.5%	80.0%
27	52.5%	50.0%
28	95.0%	90.0%
29	50.0%	47.5%
30	52.5%	55.0%
Average	(71.2 ± 4.0)%	**(71.1 ± 3.8)%**

**Table 3:** Classification accuracy for Moverio Dataset in terms of mean and standard uncertainty across all the subjects.

Method/Subject	Traditional Method	Proposed Method
1	15.0%	60.0%
2	20.0%	85.0%
3	35.0%	30.0%
4	25.0%	45.0%
5	20.0%	35.0%
6	10.0%	65.0%
7	25.0%	10.0%
8	30.0%	45.0%
9	30.0%	30.0%
Average	(23.3 ± 2.6)%	**(45.0 ± 7.5)%**

## Data Availability

Data are contained within the article.
